# Complete chloroplast genome sequence of *Convolvulus arvensis*

**DOI:** 10.1080/23802359.2021.1915202

**Published:** 2021-05-31

**Authors:** Zhiqiang Wang, Hui Song, Dechun Jiang

**Affiliations:** a Institute for Advanced Study, Chengdu University, Chengdu, China; b Teachers Training School, Baiyin, China; c CAS Key laboratory of Mountain Ecological Restoration and Bioresource Utilization & Ecological Restoration and Biodiversity Conservation Key Laboratory of Sichuan Province, Chengdu Institute of Biology, Chinese Academy of Sciences, Chengdu, China

**Keywords:** *Convolvulus arvensis*, chloroplast genome, phylogenetic analyses

## Abstract

The complete chloroplast genome of *Convolvulus arvensis* was reconstructed by reference-based assembly using Illumina paired-end data. The assembled plastome is 153,234 base pairs (bp) in length, including a pair of inverted repeat regions (IRs) of 22,662 bp each, a large single-copy region (LSC) of 89,059 bp and a small single-copy region (SSC) of 19,651 bp. A total of 115 genes were predicted from the chloroplast genome, including 74 protein coding genes, 37 tRNA genes and 4 rRNA genes. The overall GC content of *C. arvensis* chloroplast genome was 37.7%. Phylogenetic analysis with several reported chloroplast genomes showed that *C. arvensis* is closely clustered with *Operculina macrocarpa*. The complete chloroplast genome of *C. arvensis* provides new insight into the evolutionary and genomic studies of Convolvulaceae.

The perennial herb *Convolvulus arvensis* is widely distributed in the temperate regions of the world. It is a common stubborn weed in croplands. Meanwhile, it is also widely used as a traditional Chinese medicine. To date, the phylogenetic position of *C. arvensis* in the Convolvulaceae is still unclear. In this study, we first reported the complete chloroplast (cp) genome of *C. arvensis* and reconstructed a plastome phylogeny for the Convolvulaceae.

The mature and healthy leaves of a single individual of *C. arvensis* was sampled from Yinchuan Botanical Garden in Ningxia, NW China (38.42062°N, 106.177704°E). The voucher specimen was deposited in the Herbarium of Sichuan University (accession number: QTPLJQ14382553). The total genomic DNA was extracted from silica gel dried leaves using a modified CTAB method (Doyle and Doyle 1987) and sequenced based on the Illumina pair-end technology. The filtered reads were assembled using the program NOVOPlasty (Dierckxsens et al. 2017) with complete cp genome of *Ipomoea batatas* as the reference (GenBank accession no. NC_026703). The assembled cp genome was annotated using Plann (Huang and Cronk 2015), and the annotation was corrected using Geneious (Kearse et al. 2012). To examine the phylogenetic position of *C. arvensis*, a multiple sequence alignment (MSA) analyses was performed using MAFFT v7.313 (Katoh and Standley 2013) based on six cp genomes in the Convolvulaceae. Finally, a maximum likelihood (ML) tree was constructed by RAxML v8.2.11 (Stamatakis 2006) with 500 bootstrap replicates based on the alignments, using *Cuscuta exaltata* as outgroup.

The complete cp genome of *C. arvensis* was a circular molecular genome with a size of 153,234 bp in length, which presented a typical quadripartite structure containing two inverted repeat (IR) regions of 22,662 bp separated by the large single-copy (LSC) region of 89,059 bp and small singlecopy (SSC) region of 19,651 bp. The cp genome consists of 115 genes including 74 protein coding genes, 37 tRNA genes, and 4 rRNA genes. The overall GC content was about 37.7%. In the plastome phylogeny, *C. arvensis* shows the closest genetic relationship to *Operculina macrocarpa*. (98% bootstrap support) (Figure 1). The *C. arvensis* cp genome can be further used for population genomic studies, phylogenetic analyses, genetic engineering studies of Convolvulaceae.

**Figure 1.**
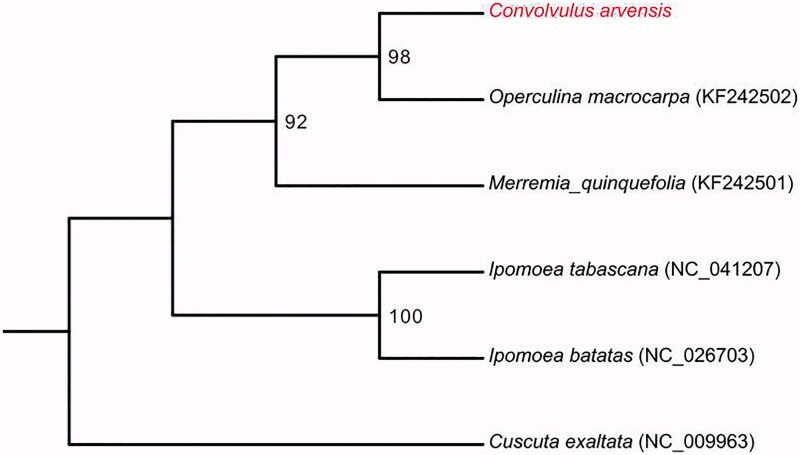
Phylogenetic relationships of six species based on chloroplast genome sequences. Bootstrap support is indicated for each branch.

## Data Availability

The data that support the findings of this study are openly available in NCBI GenBank database at https://www.ncbi.nlm.nih.gov/ with the accession number is MW054627 or available in [figshare.com] at https://doi.org/10.6084/m9.figshare.13025090. Raw sequencing reads used in this study have been deposited in the SRA database of NCBI under accession number SRR12936120.
